# Laser Treatment to avoid Trabeculectomy and to recover the Aqueous Outflow after Iris Incarceration in a Patient with NPDS

**DOI:** 10.5005/jp-journals-10008-1134

**Published:** 2013-01-15

**Authors:** Alfonso Vasquez Perez, Jorge Loscos Arenas, Julio De La Camara Hermoso

**Affiliations:** Assistant Professor, Department of Ophthalmology, Germans Trias I Pujol University Hospital, Catalonia, Spain; Associate Professor, Department of Ophthalmology, Germans Trias I Pujol University Hospital, Catalonia, Spain; Professor, Department of Ophthalmology, Germans Trias I Pujol University Hospital, Catalonia, Spain

**Keywords:** Glaucoma, Gonioscopy, Intraocular pressure, Trabeculectomy.

## Abstract

The advantages of the nonpenetrating deep sclerectomy (NPDS) compared with the trabeculectomy are based on faster recovery and lower incidence of complications. The incarceration of the iris at the trabeculo-Descemet's membrane is one complication of NPDS and leads frequently to the reoperation of the patient.

We report one case operated of NPDS with iris incarceration post-Nd:YAG goniopuncture in which we also documented spontaneous returning of the prolapsed iris during gonioscopy 3 hours after the onset of symptoms. With an argon laser iridoplasty, the iris returned completely to its position and a wide Nd:YAG laser iridotomy prevented recurrences. After this episode, the patient have always had intraocular pressure measures lower than 15 mm Hg.

**How to cite this article:** Perez AV, Arenas JL, La Camara Hermoso JD. Laser Treatment to avoid Trabeculectomy and to recover the Aqueous Outflow after Iris Incarceration in a Patient with NPDS. J Current Glau Prac 2013;7(1):36-37.

## INTRODUCTION

The advantage of nonpenetrating deep sclerectomy (NPDS) lies in their safety, lower incidence of complications and faster postoperative recovery. The Nd:YAG goniopuncture in NPDS increases humor aqueous outflow with a greater reduction in intraocular pressure (IOP).^1,2^ Although the goniopuncture is described as a safe and effective technique, it also has been reported to have associated complications, such as choroidal detachment, synechiae and incarceration of iris that carries an increased risk of failure and often requires new surgical intervention as a trabeculectomy.^1,3^

## CASE REPORT

**Figs 1A to F:**
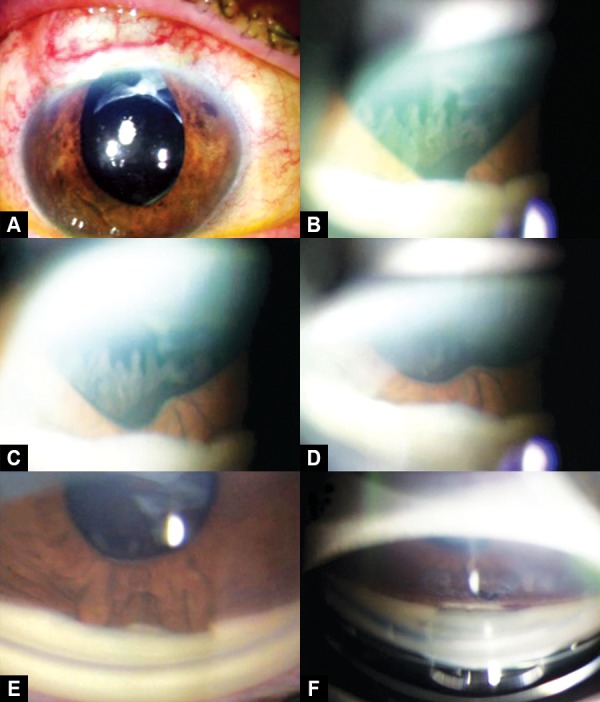
(A) Initial aspect showing a wide corectopia, (B) total iris incarceration on gonioscopy, (C, D and E) gradually returning of the iris to its normal position after a gonioscopy lens massage, (F) final aspect after laser iridoplasty and iridotomy

**Fig. 2:**
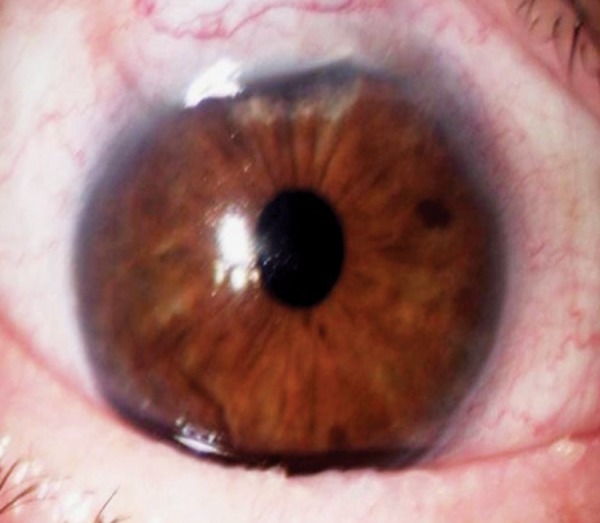
Final aspect 2 weeks after treatment with a good aspect of the filtering bleb

A 71-year-old man, with primary open-angle glaucoma and medical treatment failure in both eyes underwent a NPDS in the right eye. On the first postoperative day, the IOP was 6 mm Hg but 2 months later, the IOP increased to 18 mm Hg and an Nd-YAG goniopuncture was performed (7 pulses with 2 mJ). One week later, the patient spontaneously had sudden episode of pain and blurred vision without history of trauma. Three hours later at urgency, the examination revealed a wide corectopia with an IOP of 40 mm Hg. The diagnosis of iris incarceration was suspected and a topical treatment was initiated with a drop of pilocarpine (20 mg/ml), timolol (2.5 mg/ml) and brimonidine (2 mg/ml). Acetazolamide (250 mg) orally was also given. Two hours later, the IOP decreased to 25 mm Hg and then by gonioscopy a total prolapse of the iris by the sclerostomy was confirmed. Surprisingly during gonioscopy, almost whole prolapsed iris was pulled away gradually from trabeculodescemetic membrane (TDM) by performing a gentle massage with the gonioscopy lens (Ocular^®^ Magna View Gonio Lens) (Figs 1A to F). The treatment continued with an argon laser iridoplasty (15 hits: 200 mW, 200 ms, 300 microns) which achieved the complete retraction of the residual iris to its normal position was and the residual synechiae were released with Nd:YAG laser. Finally, we performed a broad iridotomy with Nd:YAG laser which was widened 1 week later (Fig. 2). We prescribed topical pilocarpine (20 mg/ml) and dexamethasone (1 mg/ ml) both three times daily during 1 week. Two weeks later, the IOP was 14 mm Hg without medical treatment. There were no recurrences of iris incarcerations after 6 months and the IOP had been at levels lower than 15 mm Hg without medical treatment.

## DISCUSSION AND CONCLUSION

We present one case of iris incarceration after laser-goniopuncture in a patient operated of NPDS. Vuori reported this complication in up to 10% of total post-goniopuncture cases. In a longer series of cases (258 eyes) reported by Anand, this complication was present in 13% of the patients who underwent goniopuncture becoming the most common complication associated with Nd:YAG laser goniopuncture.^1,4^ Although this complication is reported usually after laser goniopuncture, it has also been reported in patients without prior laser goniopuncture.^1,3,5^ The exact mechanism that causes iris prolapse is not known, some authors propose that increases in the IOP secondary to trauma, even subtle (as rubbing eyes) would be the cause.^1^ It may also occur spontaneously as a result of the differential pressure between the anterior and the posterior chamber in eyes with very important decreasing of IOP after laser goniopuncture.^1,3^ This could be the mechanism responsible in our patient because he did not have any history of trauma. Vouri reported that all patients with iris incarceration (three eyes) were treated performing trabeculectomy with iridotomy.^1^ Anand reported that trabeculectomy was carried out only in three eyes (1.9%) out of 173 patients. Iris incarceration carries an increased risk of failure of the previous NPDS, however, once recognized, it can be managed with argon and Nd:YAG laser iridoplasty and iridotomy to prevent recurrences. As a result of performing a wide iridotomy in two sessions, we did not report any recurrences.

Our patient underwent successfully to argon and Nd:YAG laser treatment and we accomplished restoration of the previous IOP normal levels without new surgery. As it was described in our case report, there are no previous publications that have documented the return of the prolapsed iris after an incarceration in NPDS. Probably, the most important factor for the successful final result was the short period of time between the onset of symptoms and the beginning of the treatment.
